# Publicly funded exome sequencing for outpatients with neurodevelopmental disorders demonstrates a high rate of unexpected findings impacting medical management

**DOI:** 10.1016/j.gimo.2023.100828

**Published:** 2023-08-04

**Authors:** Yara Nakhleh Francis, Tova Hershkovitz, Nina Ekhilevitch, Clair Habib, Sarit Ravid, Galit Tal, Mitchell Schertz, Adi Mory, Amihood Zinger, Hagit Baris Feldman, Rinat Zaid, Tamar Paperna, Karin Weiss

**Affiliations:** 1 Department of obstetrics and gynecology, Galilee medical center, Nahariya, Israel; 2 The Faculty of Medicine in the Galilee, Bar-Ilan University, Safed, Israel; 3 The Genetics Institute, Rambam Health Care Campus, Haifa, Israel; 4 The Ruth and Bruce Rappaport Faculty of Medicine, Technion-Israel Institute of Technology, Haifa, Israel; 5 Pediatric neurology unit, Rambam Health Care Campus, Haifa, Israel; 6 Metabolic Clinic, Ruth Rappaport Children’s Hospital, Rambam Health Care Campus, Haifa, Israel; 7 Child Development and Pediatric Neurology Service, Meuhedet, Tel Aviv, Israel; 8 Current address: The Genetics Institute, Tel Aviv Sourasky Medical Center Tel Aviv, Israel; 9 Community Genetics, Public Health Services, Ministry of Health, Jerusalem, Israel; 10 Sackler Faculty of Medicine, Tel Aviv University, Tel Aviv, Israel

**Keywords:** Autism, Clinical management, Consanguinity, Exome sequencing, Global developmental delay

## Abstract

**Purpose:**

Exome sequencing (ES) is a powerful tool that facilitates the diagnosis of patients with rare Mendelian syndromes. In 2018 the Israeli Ministry of Health initiated a national pilot program that funds ES for outpatients with global developmental delay (GDD). Here, we describe the 3-year impact of this program on patient care in a single tertiary hospital.

**Methods:**

From 2018 to 2020, trio ES was performed on 170 participants fulfilling Israeli Ministry of Health criteria: (1) moderate to severe GDD and (2) mild GDD with epilepsy or a major congenital anomaly. We retrospectively analyzed this cohort.

**Results:**

A diagnosis was achieved in 74 individuals (43%). There were 82 clinically significant variants, the majority being novel. Consanguinity was reported in 22% and was not associated with a higher diagnostic rate. The presence of autism spectrum was associated with a lower diagnostic rate of 8/33 (24%). Autosomal dominant inheritance was identified in 14% of participants, and the parental phenotype ranged between fully affected and asymptomatic. Among the diagnosed participants, 16% had an unexpected diagnosis that did not fit the typical clinical presentation. In 9%, the diagnosis changed short-term active clinical management, in 19%, the surveillance recommendations, and in 23%, the family-focused outcomes.

**Conclusion:**

The introduction of a national program that funds ES for GDD has transformed patient care, leading to a significant effect on medical management and treatment. The high rate of an unexpected inheritance mode and variable phenotypes emphasizes the diagnostic complexity of neurodevelopmental disorders and the strength of a non-targeted approach.

## Introduction

A child with a neurodevelopmental disorder is among the most frequent referrals to pediatric genetic clinics worldwide. The genetic workup of such children has advanced significantly over the past decade to incorporate extensive genomic testing, including exome sequencing (ES) and genome sequencing (GS). This has improved the diagnostic rate in children with isolated and syndromic intellectual disability (ID) to approximately 30% and 50%, respectively.^1^, ^2^, ^3^

Reaching a diagnosis has many advantages, such as providing an end to the diagnostic odyssey and enabling family planning. Still, in some cases, it can direct new treatment or follow-up recommendations significantly affecting patient care.^4^ Accordingly, several countries have implemented public funding programs for ES or GS in children with global developmental delay (GDD) or ID. The Israeli Ministry of Health (IMOH) implemented a pilot program for ES in 2018.^5^ The program funds trio ES for patients with neurodevelopmental disorders (NDD) after a non-diagnostic basic genetic workup.

Here, we report the effect of publicly funded ES performed in a tertiary center in the North of Israel on the clinical management of outpatient pediatric neurodevelopmental patients. We observed a high diagnostic yield and a high rate of novel variants significantly affecting patient management. In addition, we demonstrate a high prevalence of unexpected diagnoses, which expand the clinical spectrum of several conditions, including *CACNA1A*, *NFIX*, *SYNJ1,* and *PTPN11* related disorders. Furthermore, we identify a high rate of unexpected autosomal dominant inherited variants, with implications for parental care and pregnancy planning.

## Materials and Methods

### Participants

We retrospectively evaluated the electronic health records of 170 families that underwent ES in our institution through the IMOH pilot project from 2018 to 2020. Our pediatric genetics clinic is situated in a tertiary center in Northern Israel and serves ethnically diverse populations, including populations with a high rate of endogamy and consanguinity. We receive referrals from both outpatient neurodevelopmental pediatricians working in child developmental centers and neurologists or metabolic specialists in our children’s hospital. Patients are evaluated by certified pediatric geneticists, a process that includes the collection of detailed family and medical history, and a physical examination. Information on ethnicity was based on self-reporting and was included to identify differences between ethnic groups in our cohort. Cognitive evaluation is performed by child developmental psychologists/neurodevelopmental pediatricians located within the referring child developmental center using standardized assessment tools such as the Mullen scale, CAT/CLAMS for early learning, the Wechsler Intelligence Scale for Children, and others, to establish the developmental/intelligence quotient (DQ/IQ). The standard genetic test workup for children with neurodevelopmental disorders includes a chromosomal microarray and *FMR1* testing. In some cases, we test specific founder pathogenic variants known in the patient’s community or refer to single-gene testing where appropriate. All these tests are covered through Israeli national health insurance. ES is a second-tier test, currently available through the IMOH pilot project for probands meeting the following criteria:

1. Moderate to severe developmental delay or ID (DQ or IQ <55).
2. Mild developmental delay or ID (DQ or IQ < 70) combined with epilepsy or a major congenital anomaly including microcephaly <−2SD.

Eligible families consented to ES testing, including disclosing secondary findings as recommended by the American College of Medical Genetics and Genomics. In families identified with an autosomal dominant disease-causing variant, the clinical geneticist performed a clinical evaluation (history and physical examination) in the heterozygous parent.

### ES and analysis

Sequencing was performed at 2 certified external laboratories on a Novaseq 6000 platform (Illumina) using 1 of 3 capture kits: the Twist Human Core Exome Kit (Twist), the Agilent SureSelect Clinical Research Exome Capture Enrichment kit (Agilent Technologies), and IDT xGen Exome Research Panel V2 Kit (Integrated DNA Technologies). The average variant depth per sample was, in most cases, above 100X, and RefSeq genes were covered at 20X depth in >94% of the regions. Bioinformatic analysis was performed at RGI (Rambam Genetics Institute). Mapping of the obtained reads to the reference genome (build GRCh37/hg19), variant calling, and annotation were done using the Genoox data analysis platform/Franklin. (Genoox Ltd). Variants were prioritized based on the inheritance mode, the relation to the phenotype, the predicted effect on the protein, and minor allele frequency below 1% in general population databases and our internal database of over 2000 cases. Human Phenotype Ontology (HPO) terms were documented and incorporated into the analysis platform. For some cases, specific gene lists matching the differential diagnosis were also reviewed. A medical geneticist and a clinical molecular geneticist analyzed each case. Variants were interpreted and classified according to the American College of Medical Genetics and Genomics guidelines^6^ and Clingen. The strength of the PP3 criteria was based on Pejaver et al,^7^ the PP1 criteria was based on Jarvik et al,^8^ and the PM1 criteria was based on a review of the literature and the dolphin tool https://dolphin.mmg-gbit.eu/main.

### Effect of genetic diagnosis on patient care

For the 170 families that underwent ES in our institution, we collected demographic and clinical information on each family, including the age at referral, ethnic descent, the reason for referral, clinical findings in HPO terms, referring physician (outpatient or inpatient), the presence of ongoing pregnancy, and the genetic diagnosis. As described by Manickam et al,^4^ for cases with a diagnostic finding, we documented the effect on short-term active clinical management (treatment modifications) and long-term clinical management (ordering additional tests, recommendations for surveillance, and referrals to specialists). Surveillance with ophthalmology and audiology specialties is recommended for all our patients with developmental delay and therefore was not considered an effect on management. Furthermore, for each case, we documented the immediate reproductive outcomes and family-focused outcomes, including cascade genetic testing, referral to specialists, or changes in clinical management. Diagnostic cases were defined by the presence of a pathogenic or likely pathogenic variant that the medical team interpreted as diagnostic for the proband.

## Results

**Figure 1:**
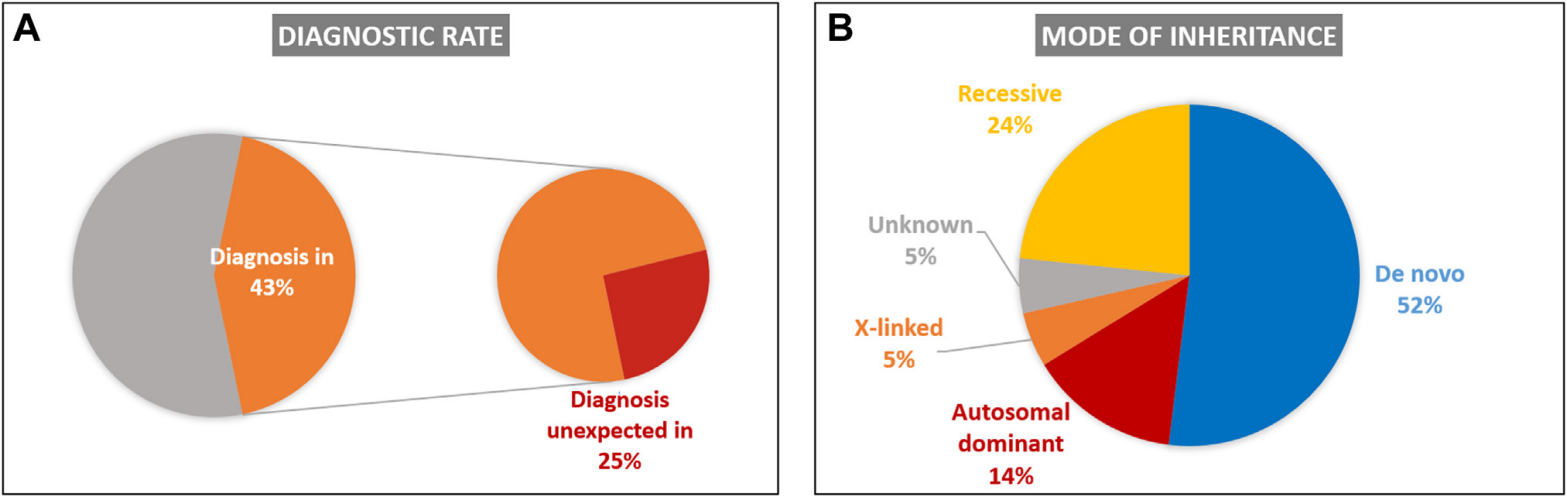
**Diagnostic rate and mode of inheritance.** A. The diagnostic yield was 75 of 170 (43%). Out of solved cases, 19 (25%) had an unexpected diagnosis with non-matching phenotypes, unexpected inheritance mode, or effect on medical treatment. B. Mode of inheritance in diagnostic cases. Unknown refers to heterozygous variants detected in non-trio ES.

The study included 170 participants between the age of 1 week and 26 years (Table 1). The average age was 5.7 years and the median was 3.5 years. The cohort included a diverse population of various ethnic backgrounds with a high rate of endogamy and consanguinity (22%) typical to the Northern Israeli population. The main clinical features are described in Table 1, and detailed features in HPO terms in Supplemental Table 1. The diagnostic yield was 43% (Figure 1A). A total of 82 pathogenic or likely pathogenic variants were identified, most of which were novel (Supplemental Table 1). A dual diagnosis explaining the phenotype was present in 3 participants (1.8%). There was no statistically significant difference in the diagnostic rate in the presence or absence of consanguinity. The presence of moderate to severe developmental delay, epilepsy, microcephaly, and congenital anomalies was not associated with a higher yield; however, autism spectrum was associated with a lower diagnostic rate of 8/33 (24%) (*P* value .021) (Table 1). The most prevalent mode of inheritance was de novo dominant (52%), followed by recessive (24%), autosomal dominant (14%), and X-linked recessive (5%). Four additional probands had suspected de novo variants, but testing of both parents was not possible, and the inheritance mode was reported as “unknown” (Figure 1B). In line with the high rate of endogamy and consanguinity, most recessive cases were homozygous recessive (68%). Among consanguineous families with a diagnosis, 58% had a homozygous recessive variant, and 35% had a de novo dominant etiology.

**Table 1:** Study cohort

Study Group	Diagnostic *n* = 74	All participants *n* = 170	*P* Value
Gender			
Male	40 (54%)	103 (61%)	NS
Female	34 (46%)	67 (39%)	
Consanguineous marriage			
Yes	16 (23%)	38 (22%)	NS
No	53 (77%)	132 (78%)	
Age			
0-1	20 (27%)	40 (23%)	NS
2-5	29 (39%)	73 (43%)	
6-12	15 (20%)	33 (20%)	
13-20	6 (8%)	20 (12%)	
Older than 20	4 (6%)	4 (2%)	
Ethnicity			
Ashkenazi Jew	17 (25%)	47 (28%)	NS
Other Jew	18 (26%)	41 (24%)	
Christian Arab	2 (3%)	3 (2%)	
Muslim Arab	24 (35%)	65 (38%)	
Druze	4 (5.5%)	7 (4%)	
North European	4 (5.5%)	7 (4%)	
Clinical features			
Moderate to severe GDD/ ID	48 (65%)	109 (64%)	NS
Autism spectrum disorder	8 (11%)	33 (19%)	.021
Epilepsy	23 (31%)	58 (34%)	NS
Microcephaly	23 (31%)	46 (27%)	NS
Major congenital anomaly	22 (30%)	46 (27%)	NS
Medical referral source			
Hospital	30 (40%)	61 (36%)	NS
Community clinics	44 (60%)	109 (64%)	

*ID,* intellectual disability; *GDD,* global developmental delay; *NS*, not significant.

**Figure 2:**
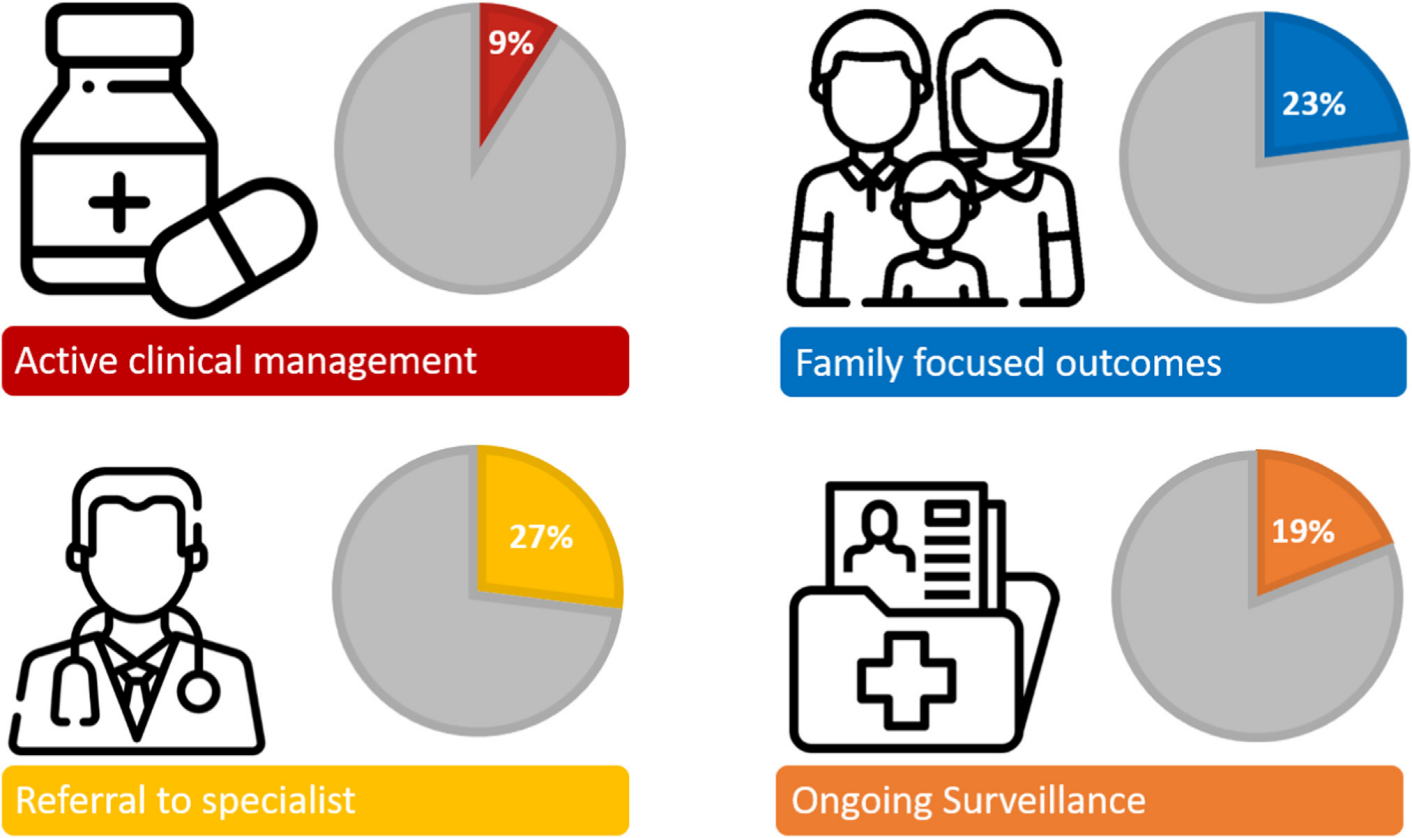
**The effect of ES on patient management.** Note: This figure has been designed using images from Flaticon.com

The most common single diagnosis was KBG syndrome (4% of diagnosed cases). Other recurrent diagnoses with locus heterogeneity were Joubert syndrome (5%) and Rasopathies (5%). The diagnosis changed short-term active clinical management in 7 participants (9%) (Table 2). It led to a high rate of long-term clinical management modification, including referral to specialists other than ophthalmology (27%) and new recommendations for surveillance other than audiology (19%) (Figure 2). Among solved cases, 12 participants (16%) had an unexpected diagnosis that did not fit the typical clinical presentation reported in the literature (Table 3). Seven families (9%) had an unexpected inheritance mode mostly autosomal dominant inherited from an unaffected or mildly affected parent (Table 4). Family-focused outcomes, with clinical impact on family members, were present in 16 cases (23%) (Figure 2).

**Table 2:** Diagnoses that led to change in treatment

Gene	Variant	Syndrome	Treatment
*ALODB*	NM_000035.3:c.448G>C; p.(Ala150Pro)	Hereditary Fructose intolerance	Dietary change
*CACNA1A*	NM_001127222.1:c.4979G>A; p.(Arg1660His	Episodic ataxia type 2	Acetazolamide for acute ataxia episode
*KCNT1*	NM_020822.3:c.862G>A; p.(Gly288Ser)	Epileptic encephalopathy, early infantile type 14	Quinidine
*ACSF3*	(ACSF3):c.1470G>C; p.(Glu490Asp)	Combined malonic and methylmalonic aciduria	Hydroxycobolamin discontinued
*MAGEL2*	NM_019066.4:c.1912C>T; p.(Gln638∗)	Schaaf-Yang syndrome	Growth hormone treatment
*IFIH1*	NM_022168.4:c.2336G>A; p.(Arg779His)	Aicardi-Goutieres Syndrome 7	Jak inhibitors
*KAT6A*	NM_006766.5:c.1951_1954del; p.(Pro651fs)	Arboleda-Tham syndrome	Carnitine

**Table 3:** Unexpected diagnoses with non-matching phenotypes at presentation

Gene	Variant	Syndrome	Non-matching features
*ALODB*	NM_000035.3:c.448G>C; p.(Ala150Pro)	Hereditary Fructose intolerance	No episodes of vomiting or hypoglycemia
*KIAA0586*	NM_001244189.1:c.2582C>T; p.(Pro861Leu/ c).428del; p.(Arg143Lysfs∗4)	Joubert syndrome	Epilepsy, normal brain MRI^a^
*KAT6A*	NM_006766.5:c.1951_1954del; p.(Pro651fs)	Arboleda-Tham syndrome	Encephalomalacia
*CACNA1A*	NM_001127222.1:c.4979G>A; p.(Arg1660His)	Episodic ataxia type 2	Global developmental delay, ventriculomegaly^b^
*KCNQ2*	NM_172107.3:c.472dup; p.(Arg158Lysfs∗15)	Benign familial neonatal epilepsy	Moderate global developmental delay, autism spectrum^c^
*SYNJ1*	NM_003895.3:c.772C>T; p.(Arg258Trp)	Developmental and epileptic encephalopathy 53	Mild intellectual disability, childhood-onset seizures
*SZT2*	NM_015284.3:c.5949_5951del; p.(Val1984del)	Developmental and epileptic encephalopathy 18	Cerebral palsy, childhood-onset seizures
*NFIX*	NM_001365902.1:c.273C>A; p.(Phe91Leu)	Malan syndrome/Sotos-like syndrome	No overgrowth, normal head circumference
*PTPN11*	NM_002834.4:c.794G>A; p.(Arg265Gln)	Noonan Syndrome	Microcephaly, no dysmorphism
*GNAO1*	NM_020988.3:c.724-8G>A	Developmental and epileptic encephalopathy 17	No seizures^d^
*NKX2-1*	NM_001079668.2:c.464-9C>A	Brain-Lung-thyroid	No chorea, normal thyroid and pulmonary function
*PTPN11*	NM_002834.4:c.1492C>T; p.(Arg498Trp)	Noonan syndrome	Normal height, no dysmorphism, epilepsy

a In the revision of the brain MRI post-diagnosis a molar tooth sign was identified.

b A similar phenotype was reported by Angelini et al.^9^

c Loss of function *KCNQ2* variants are usually associated with benign epilepsy.

d Seizures started after the molecular diagnosis.

**Table 4:** Unexpected autosomal dominant inheritance mode

Gene	Variant	Syndrome	Parental phenotype
*KCNT1*	NM_020822.3:c.862G>A; p.(Gly288Ser)	Epileptic encephalopathy, early infantile type 14	Unaffected
*CACNA1A*	NM_001127222.1:c.4979G>A; p.(Arg1660His)	Episodic ataxia type 2	Unaffected
*PTPN11*	NM_002834.4:c.794G>A; p.(Arg265Gln)	Noonan Syndrome	Unaffected
*CHD3*	NM_001005273.2:c.3358G>A; p.(Asp1120Asn)	Snijders Blok-Campeau syndrome	Mildly affected
*SIN3A*	NM_001145358.1:c.2684_2685dup; p.(Met896Leufs∗10)	Witteveen-Kolk syndrome	Mildly affected
*PTPN11*	NM_002834.4:c.1492C>T; p.(Arg498Trp)	Noonan syndrome	Unaffected
*SPEN*	NM_015001.3:c.7328del; p.(Glu2443Glyfs∗17)	Radio-Tartaglia syndrome	Unaffected

## Discussion

We aimed to evaluate the effect of publicly funded ES on the management of ambulatory patients with NDDs referred to our center. Our cohort is unique as it provides information on individuals seen in a real-life outpatient setting with predefined criteria for ES testing. Furthermore, our study provides information on long-term follow-up of 2 to 4 years after testing regarding the diagnostic yield and effect on management. We demonstrate a high diagnostic yield for ES as a second-tier test (43%), likely explained by the predefined selective criteria^5^ and the long-term follow-up.^10^ Nine of the 74 solved cases (12%) were resolved several weeks-months after the first analysis through segregation studies, parental phenotyping, gene matching tools,^11^, ^12^, ^13^, ^14^ or re-review of the current literature on candidate genes.^15^ This was achieved by a tiered analysis approach that includes a search for known disease-causing genes, as well as candidate genes matching a de novo or recessive inheritance mode. In addition, ongoing communication between clinicians and bioinformaticians in our center facilitated some of the phenotype and segregation-driven diagnoses. This process was previously shown to improve the diagnostic yield.^16^^,^^17^

Although our study population has a high rate of endogamy and consanguinity, the most common inheritance mode was de novo dominant. Interestingly, 35% of solved cases in consanguineous families had a de novo variant explaining the phenotype, emphasizing the importance of trio ES for NDDs in such populations. Compared with previous studies,^5^^,^^18^^,^^19^ we had a high rate of cases diagnosed with autosomal dominant inherited variants (14%), and interestingly, some of the heterozygous parents were identified as asymptomatic by the clinical geneticist. Pathogenic variants in *PTPN11* NM_002834.4:c.1492C>T; p.(Arg498Trp) and c.794G>A; p.(Arg265Gln) were identified in 2 probands and their unaffected parents. In both families, probands did not have typical facial features for Noonan syndrome or heart defects. In 1 case, the child was referred for mild developmental delay, microcephaly, and failure to thrive and in another case, for mild developmental delay and epilepsy. The p.(Arg265Gln) variant in *PTPN11* was previously reported with milder Noonan spectrum.^20^ In the Genome Aggregation Database, it is reported in 8 individuals, which may indicate incomplete penetrance, consistent with the family described here. In contrast, the p.(Arg498Trp) in *PTPN11* was reported in association with Noonan syndrome with multiple lentigines^21^ and seen only once in Genome Aggregation Database, supporting the highly variable clinical presentation of various *PTPN11* pathogenic variants. Another surprising finding was the asymptomatic heterozygous mother of a child with epileptic encephalopathy and intractable seizures secondary to the previously reported pathogenic variant in *KCNT1* NM_020822.3:c.862G>A; p.(Gly288Ser). The p.(Gly288Ser) variant is one of the main recurrent variants in KCNT1-related disorder and is associated with its severe form; epilepsy of infancy with migrating focal seizures. Our finding supports previous reports on incomplete penetrance in this gene, and now, we demonstrate it specifically for this presumably severe variant.^22^ Similarly, incomplete penetrance and variable expressivity, including isolated ID, were previously reported by Angelini et al in a family with a pathogenic variant in *CACNA1A*.^9^ Here, we identified another family with a recurrent *CACNA1A* pathogenic variant NM_001127222.1:c.4979G>A; p.(Arg1660His), in a proband with GDD, ventriculomegaly, hypotonia, and episodic ataxia. His heterozygous mother was unaffected but her father was reported with episodic ataxia. Finally, we identified pathogenic variants in *CHD3* NM_001005273.2:c.3358G>A; p.(Asp1120Asn), *SIN3A* NM_001145358.1:c.2684_2685dup; p.(Met896Leufs∗10), and *SPEN* NM_015001.3:c.7328del; p.(Glu2443Glyfs∗17) in mildly affected or unaffected parents supporting variable expressivity and familial cases with these conditions. In all cases, the parents were heterozygous for the pathogenic variant, which is less suggestive of somatic mosaicism.

Interestingly, in 16% of solved cases, the phenotype did not match the genetic diagnosis. Differences between the participant’s phenotype and the classic presentation included the presence of the extreme end of the phenotype with both milder or more severe manifestations, non-typical brain imaging results, or symptoms not frequently reported in the condition. In some cases, but not in all, follow-up phenotyping resolved the mismatch (Table 3). Examples for non-matching phenotypes included a child with GDD, mild dysmorphism, and a de novo likely pathogenic variant in *NFIX* NM_001365902.1:c.273C>A; p.(Phe91Leu) without signs of overgrowth, and a 13-year-old with a homozygous *SYNJ1* variant NM_003895.3:c.772C>T; p.(Arg258Trp) and an intermediate atypical phenotype of late childhood-onset epilepsy and mild developmental delay without parkinsonism. This variability in phenotypes is a well-recognized phenomenon in rare Mendelian diseases^23^ and could be related to variant properties, genetic and environmental modifiers, or additional undetected diagnoses. This emphasizes the challenges in exome data analysis and the importance of not filtering out pathogenic variants based on non-matching phenotypes.

Our study is one of the largest providing information on the effect of ES on the management of outpatients with chronic neurodevelopmental disorders.^4^ In a meta-analysis on the clinical utility of ES/GS in various in cohorts, a change in active clinical management was observed in 8%.^4^ We show similar results in a cohort of outpatients referred for developmental delays supporting the use of ES/GS with our predefined criteria. The most pronounced active clinical management effect was better control of symptoms. Examples include Quinidine significantly decreased seizure frequency in a child with KCNT1-related epilepsy, Switching to a low fructose diet improved severe growth delay in a child with a dual diagnosis of hereditary fructose intolerance and Coffin-Siris syndrome, and Carnitine improved gastrointestinal symptoms in a child with KAT6A-related disorder. In terms of the effect on long-term clinical management, we show a relatively high effect compared with Manickam et al with new recommendations for surveillance in 19% and new referrals to specialists in 27% of solved cases. Our study also provides information on family-focused outcomes, which were as high as 23%, potentially because of the high rate of autosomal dominant inheritance. Recommendations for family planning were provided in all diagnosed cases, leading to prenatal diagnostic testing or preimplantation genetic testing resulting in the birth of healthy siblings in several of the families.

In summary, our results provide real-life evidence of the significant utility of publicly funded ES in the outpatient setting directing short-term and long-term management. The high rate of an unexpected inheritance mode and variable phenotypes emphasizes the diagnostic complexity of GDD influencing diagnostic pipelines. Furthermore, our study demonstrates the advantage of using a non-targeted approach such as ES, with parental testing, and close communication between clinicians and diagnostic laboratories to achieve a higher diagnostic rate.

## Data Availability

Raw data are available upon request.

## ORCID

Karin Weiss: https://orcid.org/0000-0003-0998-810X

## Conflict of Interest

The authors declare no conflicts of interest.

## Additional Information

Appendices 3
